# Endothelial Damage in Sepsis: The Importance of Systems Biology

**DOI:** 10.3389/fped.2022.828968

**Published:** 2022-03-09

**Authors:** Jaime Fernández-Sarmiento, Luregn J. Schlapbach, Lorena Acevedo, Carolina Ramírez Santana, Yeny Acosta, Ampudia Diana, M. Monsalve, Joseph A. Carcillo

**Affiliations:** ^1^Department of Pediatrics and Intensive Care, Fundación Cardioinfantil-Instituto de Cardiología, Universidad de La Sabana, Escuela de Graduados CES, Bogotá, Colombia; ^2^Department of Paediatric Critical Care Research Group, The University of Queensland and Queensland Children's Hospital, Brisbane, QLD, Australia; ^3^Department of Paediatric Critical Care, Queensland Children's Hospital, Brisbane, QLD, Australia; ^4^Department of Paediatric Critical Care, University Children's Hospital Zurich and University of Zurich, Zurich, Switzerland; ^5^Center for Autoimmune Diseases Research (CREA), School of Medicine and Health Sciences, Universidad del Rosario, Bogotá, Colombia; ^6^Department of Critical Care Medicine and Pediatrics, University of Pittsburgh School of Medicine, Pittsburgh, PA, United States

**Keywords:** endothelium, glycocalyx, inflammation, septic shock, translational

## Abstract

The early diagnosis and appropriate stratification of sepsis continues to be one of the most important challenges in modern medicine. Single isolated biomarkers have not been enough to improve diagnostic and prognostic strategies and to progress toward therapeutic goals. The information generated by the human genome project has allowed a more holistic approach to the problem. The integration of genomics, transcriptomics, proteomics and metabolomics in sepsis has allowed us to progress in the knowledge of new pathways which are pathophysiologically involved in this disease. Thus, we have understood the importance of and complex interaction between the inflammatory response and the endothelium. Understanding the role of important parts of the microcirculation, such as the endothelial glycocalyx and its interaction with the inflammatory response, has provided early recognition elements for clinical practice that allow the rational use of traditional medical interventions in sepsis. This comprehensive approach, which differs from the classical mechanistic approach, uses systems biology to increase the diagnostic and prognostic spectrum of endothelial damage biomarkers in sepsis, and to provide information on new pathways involved in the pathophysiology of the disease. This, in turn, provides tools for perfecting traditional medical interventions, using them at the appropriate times according to the disease's pathophysiological context, while at the same time discovering new and improved therapeutic alternatives. We have the challenge of transferring this ideal scenario to our daily clinical practice to improve our patients' care. The purpose of this article is to provide a general description of the importance of systems biology in integrating the complex interaction between the endothelium and the inflammatory response in sepsis.

## Key Points

- Systems biology allows a holistic integration of the various biological systems in inflammatory and infectious diseases.
- Endothelial damage in sepsis may be the cause or effect of severe inflammation associated with systemic infection.
- The omics provide us with very useful diagnostic and prognostic tools to better understand sepsis, and allow the development of therapeutic tools based on a comprehensive understanding of the disease.

## Introduction

Sepsis is one of the leading causes of morbidity and mortality. In 2017, it was estimated to have affected 48.9 million people worldwide, accounting for 19.7% of deaths, globally (1). It is the main cause of inpatient mortality in developed countries and costs more than 24 billion dollars per year (2). Sepsis is an unregulated host response to infection which activates mechanisms leading to cellular death, with multiple organ involvement which can lead to death. The traditional approach has employed a predominantly mechanistic view, with a relationship between the involved systems based more on ontogeny and epistemology, seeking to simplify as much as possible in order to draw conclusions and find a clinical application for the diagnostic and therapeutic tools.

This reductionist view has allowed us to understand a large part of the problem, but not the problem as a whole. Multisystem involvement is common in sepsis and is partially explained by the response of each organ to the noxa and damage triggered by the infection (3). In this regard, one of the organs which is always affected to varying degrees in sepsis is the endothelium. This thin layer of cells covering the inside surface of all blood vessels tends to be affected at different times during the natural history of many diseases, including sepsis. Genetic factors, environmental influence, the degree of severity and the host's inflammatory response to infection often determine endothelial damage and its consequences. These multiple factors and the different types of response require a more comprehensive approach, from a systems biology perspective. Systems biology or medicine is a more integrated approach which seeks to analyze interactions between the components within a level of organization (genome, transcriptome, proteome, metabolome) and then between the different levels (4, 5). To date, it has been used to interpret and better understand the pathophysiology of diseases, to search for new biomarkers and therapeutic alternatives, and to develop computational models based on biomedical informatics (5, 6).

Technological advances, computational medicine and data from the human genome project have placed systems biology at the forefront of the discovery of new biomarkers (4, 5). This has allowed a better understanding of the complex pathophysiology of sepsis and endothelial involvement, and their interesting interaction with the inflammatory response. These findings have allowed us to explore new therapeutic alternatives aimed at modulating inflammation and endothelial injury. The integrating capacity and holistic approach of systems biology in sepsis provides an understanding of the complex interaction between host and pathogen in the various pathophysiological stages involved (genomic, transcriptomic, proteomic, metabolomic) (6–8). Specifically, systems biology has allowed us to understand and delve deeper into the relationship and interaction of the endothelium with the inflammatory response in patients with sepsis. The purpose of this review is to use systems biology to integrate this interaction between the inflammatory response and the endothelium in patients with sepsis. We sought to include studies which integrated sepsis, the endothelium and systems biology to understand that the approach to this disease is integral to a continued advance toward precision medicine in sepsis.

## Systems Biology in Sepsis

Systems biology in sepsis is a field of study dedicated to determining the various complex interactions within a system with different biological levels of organization (5, 9, 10). It is characterized by a quantitative description of the biological processes which include multiple levels starting from the omics (such as the genome, transcriptome, proteome, and metabolome), and which may be simultaneously or progressively activated over time. To approach a system in this way, the data should be generated and supported by highly developed and specialized technology, using computational models, biomedical informatics and mathematical algorithms. Often, these interactions are portrayed in what is known as a “network” diagram (7, 8).

To organize and analyze the large amount of data generated by the omics, systems biology uses computational medicine to provide a comprehensive approach to the problem. An example in sepsis is the analysis of the enormous number of genes which are activated in the presence of infection and the signaling pathways which consequently magnify the inflammatory response in the endothelial cell lining (ECL) (10). At this level, type I activation (with elevated release of vasodilating substances such as nitric oxide and prostacyclin) and type II activation (with increased damage at tight junction points, cadherin) of the endothelium have been found, which lead to a significant magnification of the systemic inflammatory response, as explained further on (11).

**Figure 1:**
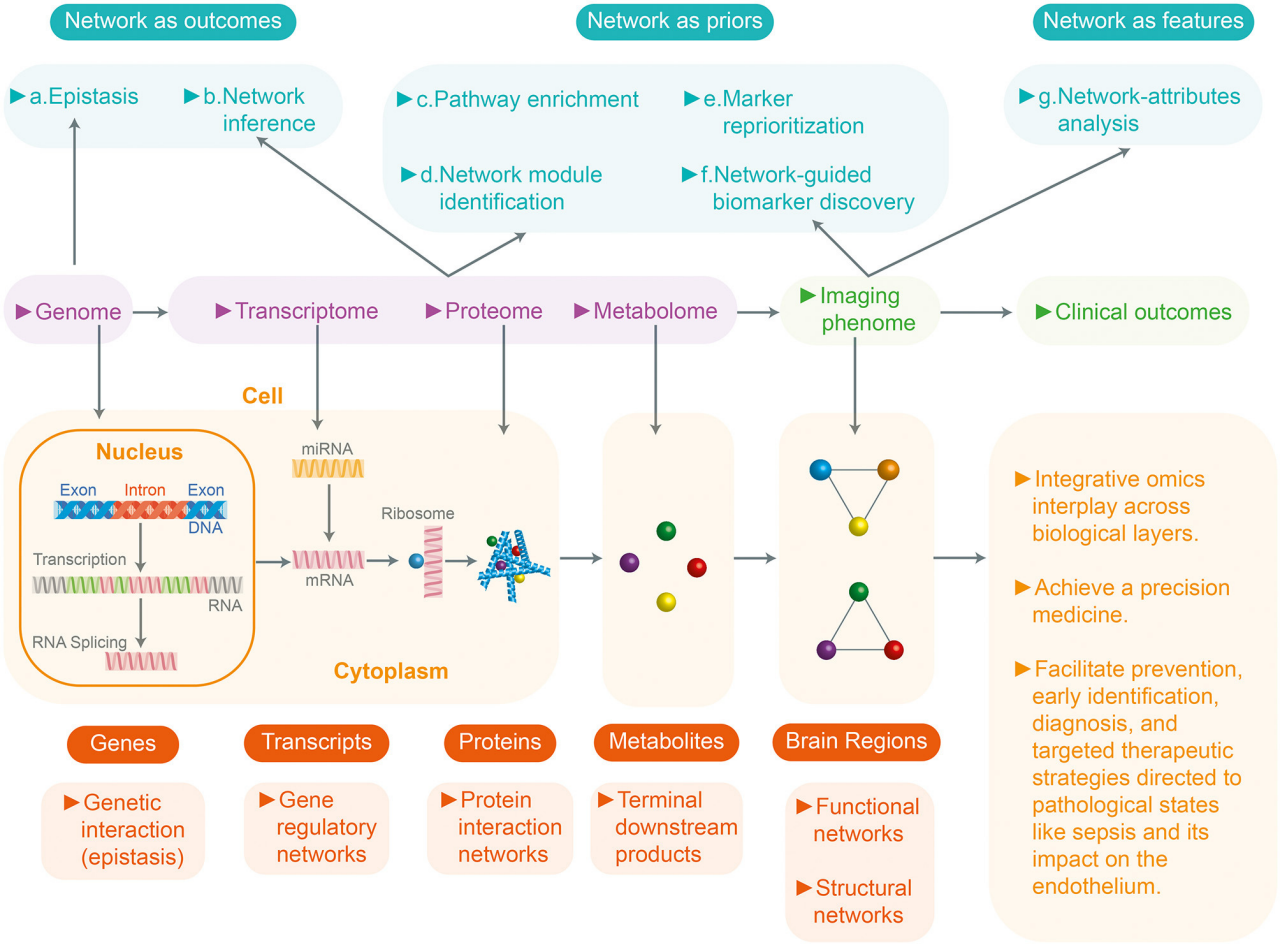
Systems biology and integration with omics and multidata in sepsis. Systems biology or medicine seeks a more integrated approach to analyze the interaction between the components of a system (genome, transcriptome, proteome, metabolome) and then the interaction between the different levels. This figure shows a review of the network approaches across multiple biological layers for the analyses of the multi-omics data in complex disease studies. Based on the role of networks, analytic approaches can be divided into three groups: (1) Networks as outcomes (explore the relationships between entities) (2) Network as prior (uses existing networks as prior knowledge to guide the analytic procedure). (3) Networks as features (analyzes the prior networks regarding their topology and attributes: nodes and edges). They can be further divided into seven subcategories (a-g). At the bottom, the four biological (genome, transcriptome, proteome, metabolome) levels and their unit of study are described. All these resulting networks of knowledge will inform precision medicine.

Systems biology in sepsis integrates these various genetic expressions with the activation of signaling pathways which release proteins that have very significant biological effects. Biomedical informatics and the various software packages available on the market are essential tools for understanding this interaction (12). The biomedical informatics process entails two basic steps: data processing and quality control analysis. Once these steps are carried out, the genomic and proteomic variables are normalized and the variables which are altered in sepsis are compared with controls, using statistical models (p value, analysis of variance, correlation, etc.) to identify the variables which are especially associated with a type of inflammatory response (7). There are many commercial and software packages available today for these analyses (Ingenuity Systems, Cytoscape, GeneGO, Partek) (8, 11, 12). These software packages integrate biological pathways from genetic expression through protein synthesis, metabolites, etc., based on the scientific literature and using common language processors. These analyses provide an understanding at the genomics level of the biological effects of the variables which magnify the individual inflammatory response in sepsis, allowing medical precision in selecting targeted therapeutic strategies (Figure 1).

This approach helps us understand why many studies in animal models do not have the same results as clinical trials (13). Animal models generally use young specimens which are subjected to a specific injury with a limited response mechanism and concentrated on a restricted group of pathways (redundant and hierarchical modules) (14). In addition, many experimental animals may be genetically related, with these findings clearly not extrapolatable to a population of sepsis patients which tends to be quite heterogeneous with regard to individual characteristics, comorbidities and environmental influence (14, 15).

## Endothelial Damage in Sepsis

**Figure 2:**
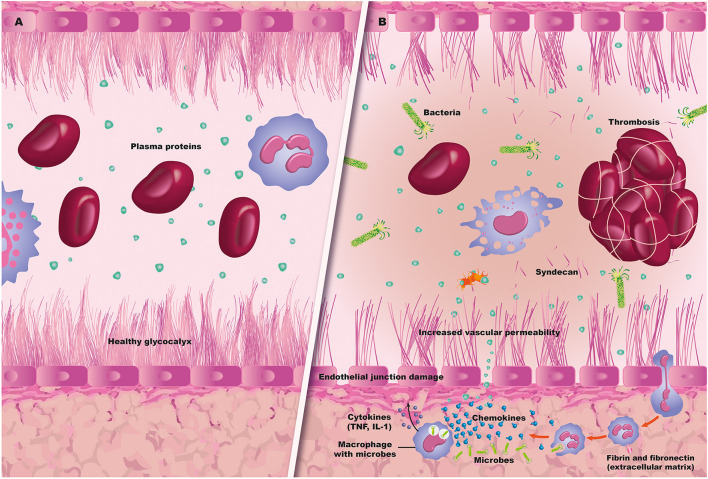
Sepsis and endothelium. **(A)** A normal blood vessel with an intact glycocalyx and endothelium. **(B)** Sepsis-related endothelial damage, with bacteremia and thrombus formation, along with capillary leak and leukocyte migration.

Endothelial activation and damage is one of the essential steps in the cascade of multiorgan involvement in patients with sepsis. Under normal conditions, the ECL has very important functions (16). It is not simply a layer of flat, elongated, polygonal cells covering the luminal surface of all blood vessels and strongly attached to the extracellular matrix. It has bidirectional functions and relationships with blood components. That is, it acts as both a signal receptor and transmitter. It registers hemodynamic changes such as pressure and shearing or friction forces, allows interaction with leukocytes and platelets, and modifies circulating chemical messengers. Thus, the old concept of a simple barrier separating the blood from the vascular wall has changed, and the ECL is considered to be the main organ for vascular regulation, with exocrine, paracrine and autocrine actions (17–19) (Figure 2).

In addition, the ECL controls vascular permeability as it maintains tight junctions (or zonula occludens), represented mainly by proteins such as claudin and occludin. It also has gap or communicating junctions (connexin 37, 40, 43), adherens junctions (mainly cadherins) and syndesmos (19). These junctions, together with the protective layer of the endothelial glycocalyx, prevent cellular migration and capillary leak, and maintain an anti-inflammatory and anti-thrombotic state. This is what has been called an “antiadherent” endothelial phenotype, preventing the formation of clots and systemic perfusion disorders.

However, endothelial activation by these stimuli is not an “*all or nothing*” phenomenon. That is, there is a broad range of presentation which depends on the host's prior conditions, comorbidities and the severity of the noxa, among others (18). In addition to pyroptosis, the ECL may experience apoptosis (under normal conditions, <0.1%) which increases the inflammatory response, favoring the paracrine action of pro-inflammatory cytokines over the induction of ICAM-1, VCAM-1, procoagulant activity and complement activation.

### Endothelial Activation and Glycocalyx Damage

When a noxa activates and damages the ECL, a series of cell responses occur which magnify the ongoing inflammatory phenomenon. The main endothelial functions affected in sepsis include altered vasoregulation, barrier function, inflammation and hemostasis (20). Among other mechanisms, this damage occurs due to endothelial glycocalyx degradation, altered nitric oxide release, inadequate regulation of reactive oxygen species (ROS) and activation of adhesion molecules. Likewise, a large amount of protease and tissue factor is released, which favors the prothrombotic state typical of patients with sepsis (13, 19, 20).

Mucous membrane and skin alterations, cytokine release and phagocyte and granulocyte activation in the presence of infectious noxa have significant repercussions on ECL integrity, leading to cytoskeleton alterations and endothelial glycocalyx degradation. The glycocalyx is a 0.2 to 0.5-micron layer anchored to the endothelial cell's cytoskeleton, and it fulfills a barrier function which prevents endothelial activation (21, 22). It is made up of proteoglycans (syndecan-1, glypicans), glycosaminoglycans (mainly heparan sulfate) and glycoproteins (23–25). It is responsible for keeping the ECL isolated from significant inflammatory activation. Shedding of the glycocalyx occurs due to the presence of oxidants, proteases, cytokines and some bacterial endotoxins (26). Tumor necrosis factor-alpha and heparanase have been found to be two main mediators which intensely damage the endothelial glycocalyx (25–28). In an animal model, Chappel et al. observed profound damage of the glycocalyx in coronary arteries induced by TNF, which could be recovered with a hydrocortisone infusion (27).

Likewise, in animal models, the release of matrix metalloproteinases during oxidative stress and inflammation has been found to directly damage the endothelial glycocalyx (29). All of this leads to, among other effects, the exposure of adhesion molecules (selectins) and activated leukocyte transmigration (integrins), with activation of hemostatic components favoring a prothrombotic and antifibrinolytic state typical of sepsis (16). In addition, mechanotransduction of the endothelial glycocalyx is lost, with nitric oxide overproduction and loss of intercellular junctions which are essential in the integration of vasomotor control, with consequent capillary leak, edema and loss of the endothelial barrier (30).

A large proportion of these phenomena occur within the first 10–20 min after the noxa, and are what has been called type I endothelial activation (Table 1). This activation occurs due to the binding of ligands such as IL-1 or TNF to G-protein-coupled receptors which activate a cascade of intracellular signals which, in addition to releasing intracellular calcium from the endoplasmic reticulum, also activate the phosphorylated myosin light chain (MLC) responsible for Weibel-Palade body exocytosis. This activation leads to the release of von Willebrand factor which facilitates platelet aggregation and a procoagulant state.

**Table 1:** Types of endothelial activation.

**Characteristic**	**Type I**	**Type II**
Onset after noxa	10–20 min	Hours
Activation	Ligand coupled to a G-protein receptor	Inflammatory cytokine (IL-1 or TNF)
Mediator	Intracellular calcium and phospholipase A release	Nuclear factor-kb
ECL products released	Prostaglandin I2, nitric oxide, von Willebrand factor	E-selectin, ICAM-1, VCAM-1, heparan sulfate, chemokine
Leukocyte recruitment	+	+++
Loss of intercellular adherens junctions	+++	++
Increased vascular permeability	+++	+++

*ECL, endothelial cell lining.

Type II endothelial activation occurs in response to inflammatory cytokines such as IL-1 and TNF. The binding of these cytokines with their receptors in the ECL initiates the activation of signaling pathways that increase transcription of NF-kb and other new proteins (10, 12). The proinflammatory response induced by these cytokines leads to the transcription of new genes and translation of new proteins; thus, type II endothelial activation tends to take longer than type I (h vs. min). Type II activation increases blood flow, as well, fostering capillary leakage of plasma proteins with increased recruitment of leukocytes to the site of inflammation (18). The sustained leakage of plasma proteins leads to an especially firm provisional matrix, often described as induration (a hard swelling), instead of the soft and transient edema characteristic of the fluid-rich leakage seen in type I activation. The hardness is due to the leakage of very large plasma proteins, like fibrinogen, which subsequently become a fibrin-rich clot (30).

## Genomics in Sepsis

Genomics is the study of the entire genome, all the organism's genes, in contrast to genetics which studies individual genes. One of the great advances for systems biology and the comprehensive approach to disease was the international human genome project. After 13 years, the three billion DNA base pairs of the human genome were able to be sequenced, estimating a total of 28,000–34,000 genes (7). These discoveries laid the functional foundation for genomic medicine, computational medicine, biomedical informatics and systems biology which have led to great advances in the knowledge of many diseases such as sepsis. However, the significant role of the individual burden in the person's response to infection had already been considered for years. In 1988, Sorensen et al. studied adopted children in Denmark to research deaths from all causes (31). They found that if the biological father had died from infection before the age of 50, the child had a 5.8 times greater relative risk of also dying from infection. These findings suggest the importance of genetics in the response to sepsis, and make genomic research particularly interesting.

### Clinical Application

The existence of a simple “sepsis gene” is not biologically plausible. The existence of genetic variations with multiple candidate genes which affect the host response to infection is more realistic. Wong et al. studied 6,934 genes and generated 10 clusters of genes regulated by the activation of systemic inflammatory response syndrome signaling pathways (32). A total of 100 genes with a strong predictive value for inflammation and systemic damage were identified. Of these 100 genes, 44 correspond to relevant signaling pathways in the adaptive immune system and were found to be related to patients with sepsis. The researchers found three sepsis response endotypes, of which endotype A had a three times greater mortality than the others (36%), and these endotypes were related to genes which magnified the adaptive immune system response, increased the expression of steroid signaling receptors and regulated zinc homeostasis (32).

Most studies researching the relationship between genetics and sepsis have focused on the identification of polymorphisms: the regular occurrence (<1%) of two or more alleles in a particular chromosome location. The most common type of polymorphism is called single nucleotide polymorphism (SNP), which consists of a substitution, deletion or insertion of a single nucleotide occurring in approximately 1 out of every 1,000 base pairs in human DNA (7). This SNP may result in a protein synthesis disorder, a change in the quantity and normal expression of proteins or an alteration in their function. An example of this phenomenon seen in clinical practice is the endothelial activation with microvascular microthrombosis found in patients with meningococcal sepsis. These patients have polymorphism of the gene which codes for tissue plasminogen activator inhibitor type 1 (PAI-1), which is considered to be a procoagulant protein, as it inhibits fibrinolysis (33–35). Hermans et al. found a 4G/4G genotype in children with meningococcal sepsis which produces high concentrations of PAI-1 and is associated with worse outcomes in these patients compared with 4G/5G or 5G/5G genotypes (33).

More recently, Wong et al. identified 100 genes associated with mortality in septic shock (36). They were able to identify 12 gene-related protein biomarkers which were biologically plausible and measurable in the blood. Five biomarkers with predictive capacity for mortality and organ damage, including endothelial damage in patients with sepsis, were selected. They determined that chemokine ligand 3 (CCL3), IL-8, heat shock protein 1B, granzyme B and matrix metalloproteinase-8 (MMP8), combined, could help identify septic patients with worse outcomes. These biomarkers were found to be associated with TP53 pathway activation, which is responsible for eliminating damaged DNA, precipitating apoptosis and modulating cellular metabolism, autophagy and NF-κB production. These biomarkers combined, known as PERSEVERE XP, could identify and stratify risk groups for mortality in septic shock.

### Advantages

Identifying a group of genes which are activated in patients with sepsis and increase the translation of proteins with specific signaling pathways could help identify therapeutic targets and risk groups (37). Endothelial and microcirculation damage in sepsis has helped identify risk groups with a greater tendency toward developing hypercoagulability and thromboses. This early identification of susceptibilities would allow early initiation of prophylactic anticoagulation in patients with an infectious noxa. Similarly, one of the major culprits of type II endothelial activation is TNF (Table 1) which, when it has an SNP in position 308, has been associated with a high degree of expression both *in vivo* and *in vitro*, and has been associated with up to four times greater mortality in studies of patients with septic shock. Early identification of this SNP would allow risk groups to be identified for targeted therapies (38). The detection of genetic susceptibility to infection, its severity and the involvement of specific organs like the endothelium not only allows targeted therapy, but also the design of clinical trials to identify specific subgroups and determine which groups have a greater or lower possibility of responding to a therapeutic strategy. This is one step closer to precision medicine in sepsis (38).

### Limitations

One of the great disadvantages of involving genomics in patients with sepsis is the availability of these diagnostic and prognostic tools for all patients. The different healthcare settings for treating septic patients (such as the emergency room, hospitalization and critical care), the severity of systemic involvement (including the microcirculation and endothelium), and the difficulty in accessing healthcare services are barriers that must be overcome in order to access genomic stratification with therapeutic implications. Likewise, the functional variants of sepsis, modifying phenotypes which alter molecular function, and an exclusive genomics approach could oversimplify and overlook other factors related to the disease which are associated with unsatisfactory outcomes. These include existent comorbidities, germ aggressiveness and resistance, and the limitations to healthcare access mentioned (especially in low- and middle-income countries). These factors do not depend on the genetic response and may be associated with mortality and unsatisfactory outcomes.

## Transcriptomics in Sepsis

This is the next level of study after genomics (3). Transcriptomics studies gene activity and its regulation. The DNA expresses its information through a process known as transcription (4). In this process, segments of the DNA sequence are used as templates representing the cell's genetic information. Thus, pre-messenger RNA (mRNA) results from transcription which, after splicing, creates a final mRNA. This mRNA is the genetic mediator which guides protein synthesis in the ribosome, according to the information stored in the DNA. Therefore, transcriptomics seeks to document this gene activity through mRNA quantification, and thus measure not only levels but also patterns of gene expression which are characteristic of various biological states, such as sepsis (39, 40) (Table 2).

**Table 2:** Advantages and limitations of transcriptomics, proteomics and metabolomics in endothelial damage in patients with sepsis.

**Biological level of study**	**Advantages**	**Limitations**
Transcriptomics Expression profiling (MicroArray)	- Creates an overall view of the transcriptome alterations. - May elucidate alterations in a signal transduction pathway.	- Low sensitivity for measuring low-expression genes - Tissue-specific expression
RNA- seq	- Unbiased approach. - Estimates abundant genes in terms of copies.	- Tissue-specific expression
miRNA	- Stable in blood. - Has biomarker potential. - Evolutionarily conserved and are often tissue or pathology specific. - The evidence suggests an important role in understanding network regulation.	- The functions are not fully understood, and the underlying mechanism continues to be a question.
Proteomics	- High sensitivity - Less restrictive than ELISA and multiplex techologies. - An unlimited number of proteins may be simultaneously analyzed. - Does not require antibody-based technologies for measuring proteins.	- Requires a lot of preprocessing - Blood proteins vary by more than 10 orders of magnitude. - Difficult quantification of low-expression proteins
Metabolomics	- High sensitivity - Sensitive to biological alterations and responds with rapid changes. - Protein quantification does not require antibody-based technologies.	- Require a lot of preprocessing - Difficult quantification of low-expression proteins - The chemical and physical properties of the metabolites vary widely.

This process of gaining access to the gene expression profile is accomplished through three types of techniques, some based on hybridization and others on sequencing: (1) microarrays, (2) RNA-seq, and (3) miRNA (1). Microarrays are a hybridization technique which uses previously identified transcripts of interest on a single microscope slide to determine which genes are active or inactive in the cell. This technique requires mRNA which is extracted from the tissue and subsequently labeled with an enzyme to create complementary DNA (cDNA), with certain fluorescent nucleotides bound to this cDNA. This labeled cDNA is placed on the microarray DNA slide so that when mRNA encounters its cDNA, they bind and create a fluorescent mark. The intensity of this fluorescence indicates the quantity of mRNA bound to cDNA, and thus this gene's activity. If, on the other hand, fluorescence is not produced, this indicates that the gene is inactive.

On the other hand, RNA-seq is a next generation mass sequencing technique (Next Generation Sequencing [NGS]) which does not require sequencing information to detect and evaluate transcription, and thus gene activity (39). The main objective of RNA-seq is to catalog each of the transcripts (RNA) expressed by a cell in a specific condition.

Finally, miRNA is a small non-protein coding RNA made up of 20–24 nucleotides which regulates gene expression by inhibiting translation or transcription of the target mRNA (41). This miRNA has an inhibitory effect on gene expression and control. Its regulating effect on protein coding genes is produced not only through inhibition of protein translocation (40) but also of mRNA degradation (41).

### Clinical Application

The miRNAs are critically involved in the innate and adaptive immune response in pathological disorders such as arteriosclerosis, diabetes and infections like sepsis (41, 42). Their effect on significant pathways such as the TNF (as miRNA 155 and miRNA 145) and IL-6 pathways (as MiRNA 21, 23, and 29) has been described (41). However, at the same time, miRNAs have been detected in various body fluids: blood, urine and sweat, allowing the creation of panels of these miRNAs in conditions such as sepsis, which suggests that circulating miRNAs could also be biomarkers in the context of sepsis (41, 43).

Chen et al., after transcriptional analysis, evaluated endothelial cell activation and damage in the adrenal microvasculature in sepsis (44), an important element given the important role of the hypothalamic-adrenal axis as well as the sympathetic-medullary system as response systems in this disease. They found activation of upregulated gene transcriptors such as chemokines (CCL2, CXCL10, CCL5), leukocyte adhesion molecules (SELP, Vcam-1), angiopoiesis molecules (PDGF), and inflammation molecules (SphK1, IL-6, PTGS2); along with gene transcripts which cause downregulation of apoptosis, autophagy and cell junction (PAPKs, RAPSN, DSC2) signaling processes (44). This evidenced a robust transcriptomic response which included pathogen recognition receptors, cytokines, chemokines, immune cell modulators and adhesion molecules (44). This transcriptomic analysis study suggests the expression of several genes which are potentially involved in endothelial cell damage in the adrenal microvasculature, and how this could be an initial step in the adrenal dysfunction which develops in many patients with sepsis, associated with high mortality.

## Proteomics in Sepsis

This is considered to be the next level in the study of biological systems after genomics and transcriptomics. It is the area of large-scale protein discovery, defined as the one responsible for quantifying and categorizing all the proteins expressed in the cell, tissue or organs (11). The focus of proteomics can be subdivided into three types: expression, structural and functional (11). Expression proteomics is responsible for enumerating the expression of all proteins present in the cells, tissues or organisms, as well as identifying the proteins which are specifically positively or negatively regulated in a disease, for use as diagnostic markers. On the other hand, structural proteomics aims at mapping proteins from their formation. Finally, functional proteomics seeks to clarify the biological function of proteins, their interactions, post-translational modifications and the definition of cell mechanisms at this molecular level (11).

### Clinical Application

As mentioned previously, Wong et al. (36) not only identified 100 genes associated with mortality in septic shock, through genomics, but were also able to follow their expression through the subsequent biological levels until they documented the presence of five biomarkers at the proteomic level: CCL3, IL-8, heat shock protein 1B, granzyme b and MMP8, which are known jointly as PERSEVERE XP, and, when found in septic patients, have worse outcomes and greater mortality from sepsis.

Likewise, other researchers have measured other inflammatory proteins which are less specific but very useful for orienting toward the severity of involvement and its possible extent (45). This is the case of C-reactive protein (CRP). This protein is an acute phase reactant from the pentraxin group which has been found to rise significantly in septic patients. Lanziotti et al. found four patterns of elevation in children with sepsis which suggest a biphasic behavior (decreasing with interventions and then rising) associated with an almost four times greater mortality (46). Similarly, serum ferritin, which is not specific to patients with sepsis either, is a protein which has been used as a biomarker for inflammatory response. Levels above 500 mg/dl have been associated with macrophage activation, increased IL-1 and IL-18, and the creation of a large quantity of inflammasomes with triggered programmed cell death (47). When these two biomarkers (CRP and ferritin) are evaluated together, clinical studies have found worse outcomes in septic patients, and they may be very useful proteins for evaluating follow up and predictors of poor outcomes (48).

## Metabolomics in Sepsis

This is the study of the metabolome, a collection of small molecules produced by the cells which are responsible for the organism's metabolic processes. There are specific and nonspecific approaches to studying the metabolome. The specific metabolomic approach is aimed at measuring a specific number of metabolites in a pathway of interest (49). This allows researchers to generate a specific biochemical question based on a hypothesis. It is important to note that it offers deeper understanding of the proposed hypothesis, but depends on robust data and prior knowledge. This type of metabolomics uses triple quadrupole mass spectrometry (50, 51) and is a highly sensitive method for the absolute quantification of metabolites, with a very low concentration. On the other hand, the nonspecific metabolomic approach is an unbiased screening method for identifying thousands of metabolites in a single sample, thus enabling exploratory studies of currently unknown metabolites (38). It uses platforms such as nuclear magnetic resonance (NMR), gas chromatography-mass spectrometry (GC-MS) and liquid chromatography-mass spectrometry (LC-MS).

### Clinical Application

Systemic inflammation has emerged as a key pathophysiological process the induces multiorgan injury and the endothelium is critical in maintaining cellular and inflammatory homeostasis. Wang Yi-Fu et al. found that conditioned meditioned medium of endothelial cells inhibited cyclooxygenase-2 and interleukin-6 expression in macrophages (52). Analysis of conditioned medium extracts by liquid chromatography-mass spectrometry showed the presence of 5-mehoxytryptophan (5-MTP). Endothelial cell-derived 5-MTP suppressed lipopolysaccharide induced inflammatory responses and signaling in macrophages and endotoxemic lung tissues. They conclude that 5-MTP is a endothelium-derived protective molecules that defend against endothelial barrier dysfunction and excessive systemic inflammatory responses (53, 54).

## Conclusions

Systems biology provides a comprehensive approach to endothelial damage associated with inflammation in patients with sepsis. Understanding the role of omics as diagnostic tools to better understand the pathophysiology of the disease can help us seek a holistic approach to the bidirectional phenomenon of inflammation and endothelial damage in infection models such as sepsis. Strategies are needed to eliminate barriers and facilitate access to all these systems medicine tools at the patient's bedside, especially in limited resource settings.

## Author Contributions

JF-S, LS, LA, CS, YA, AD, MM, and JC contributed to designing and performing the review. All authors contributed to drafting the manuscript and reviewing the final article. All authors approved the final manuscript as submitted and agree to be accountable for all aspects of the work.

## Conflict of Interest

The authors declare that the research was conducted in the absence of any commercial or financial relationships that could be construed as a potential conflict of interest.

## Publisher's Note

All claims expressed in this article are solely those of the authors and do not necessarily represent those of their affiliated organizations, or those of the publisher, the editors and the reviewers. Any product that may be evaluated in this article, or claim that may be made by its manufacturer, is not guaranteed or endorsed by the publisher.
